# Cytochalasins as Modulators of Stem Cell Differentiation

**DOI:** 10.3390/cells13050400

**Published:** 2024-02-25

**Authors:** Luca Pampanella, Giovannamaria Petrocelli, Provvidenza Maria Abruzzo, Cinzia Zucchini, Silvia Canaider, Carlo Ventura, Federica Facchin

**Affiliations:** 1 Department of Medical and Surgical Sciences (DIMEC), University of Bologna, Via Massarenti 9, 40138 Bologna, Italy; luca.pampanella2@unibo.it (L.P.); giovannam.petrocell2@unibo.it (G.P.); provvidenza.abruzzo2@unibo.it (P.M.A.); cinzia.zucchini@unibo.it (C.Z.); federica.facchin2@unibo.it (F.F.); 2 National Laboratory of Molecular Biology and Stem Cell Bioengineering of the National Institute of Biostructures and Biosystems (NIBB) c/o Eldor Lab, Via Corticella 183, 40129 Bologna, Italy

**Keywords:** cytochalasins, stem cells, mesenchymal stem cells, mesenchymal stromal cells, actin microfilaments, cytoskeleton, cell differentiation, osteogenesis, adipogenesis, chondrogenesis

## Abstract

Regenerative medicine aims to identify new research strategies for the repair and restoration of tissues damaged by pathological or accidental events. Mesenchymal stem cells (MSCs) play a key role in regenerative medicine approaches due to their specific properties, such as the high rate of proliferation, the ability to differentiate into several cell lineages, the immunomodulatory potential, and their easy isolation with minimal ethical issues. One of the main goals of regenerative medicine is to modulate, both in vitro and in vivo, the differentiation potential of MSCs to improve their use in the repair of damaged tissues. Over the years, much evidence has been collected about the ability of cytochalasins, a large family of 60 metabolites isolated mainly from fungi, to modulate multiple properties of stem cells (SCs), such as proliferation, migration, and differentiation, by altering the organization of the cyto- and the nucleo-skeleton. In this review, we discussed the ability of two different cytochalasins, cytochalasins D and B, to influence specific SC differentiation programs modulated by several agents (chemical or physical) or intra- and extra-cellular factors, with particular attention to human MSCs (hMSCs).

## 1. Introduction

Regenerative medicine is an emerging field of medical research that aims to replace, repair, or regenerate damaged tissues and organs in patients suffering from severe injuries or chronic diseases, in which the body’s regenerative responses are not sufficient [1,2]. Since the transplantation demands exceed the number of donated tissues and organs, research efforts in the field of regenerative medicine have focused on tissue engineering technology, i.e., the transplantation of stem cells (SCs) often combined with specific scaffolds (such as biodegradable 3D scaffolds) [3] or treatments (such as growth factors), as well as on other strategies that stimulate the regenerative potential of endogenous/resident cells in damaged tissues.

To achieve regenerative outcomes, the transplanted SCs can exert direct effects. In fact, they can survive, proliferate, and differentiate into the damaged or lost cell types, integrating themselves in a specific tissue niche and into the host’s circulatory system [2]. Moreover, SCs can exert an indirect effect on the resident cells by secreting paracrine and immunomodulatory factors, thus stimulating their repairing properties [4,5]. However, the amount of SCs used during transplantation procedures affects the outcome of the regenerative process, and therefore, an ex vivo expansion of transplantable SCs is necessary to obtain an adequate cell number [2]. On the other hand, SC long-term in vitro expansion may result in the accumulation of genetic damage and intra-cellular signaling pathway alterations, ultimately leading to the senescence process, a condition in which cells perform less than early-passage SCs [6]. 

Therefore, the objective of this research is to identify, first in vitro and then in vivo, new strategies to modulate the biological properties of SCs and to attempt to slow down the senescence process, preserving their innate self-renewal capacity and enhancing their differentiation potential, in order to improve their efficacy in transplantation approaches.

Many factors can modulate the differentiation capacity of SCs, such as cytochalasins and cyto-permeable mycotoxins, by modifying the actin cellular organization [7,8], which can induce a specific and different cell commitment depending on the dose and type of exposed SCs [9]. 

Cytoskeletal networks, by influencing mechano-sensing and mechano-transduction pathways [10], may affect SC fate, as can be demonstrated by changes in mechanical properties detected during specific commitments [11]. For instance, under adipogenic and chondrogenic differentiation, mesenchymal stem cells (MSCs) lose and disorganize their cytoskeletal framework, becoming softer and taking a round shape. In contrast, the osteogenic commitment is encouraged by a stiffer and well-organized cytoskeleton as well as the presence of focal adhesions [12]. 

In this review, we summarize and discuss published data about the effects of cytochalasins on the main SC differentiation programs (osteogenesis, adipogenesis, chondrogenesis, odontogenesis, myogenesis, tenogenesis, and neurogenesis), focusing on human MSCs (hMSCs). A deepened and careful reading of all articles allowed us to extrapolate data about the role of these mycotoxins in SC commitments which are modulated by several agents (chemical or physical) or intra- and extra-cellular factors. 

## 2. Methods

### Search Strategy and Study Selection 

A PubMed search was performed up to February 2024. The search strategy included (i) the MeSH terms “stem cells”, “mesenchymal stem cells”, “stromal cells”, “cytochalasins”, “osteogenesis”, “adipogenesis”, “chondrogenesis”, “odontogenesis”, “muscle development”, and “neurogenesis”, and (ii) the terms “mesenchymal stromal cells”, “myogenesis”, “cardiogenesis”, “tenogenesis”, osteogenic”, “adipogenic”, chondrogenic”, “odontogenic”, “myogenic”, “cardiogenic”, “tenogenic”, and neurogenic”. Searches were performed by either using or not using the MeSH terms. Searches without the MeSH terms allowed us to select the greatest number of articles. Therefore, the final search was performed as follows: ((stem cells) OR (mesenchymal stem cells) OR (mesenchymal stromal cells) OR (stromal cells)) AND (cytochalasins) in combination with (AND) a term referred to a specific cell differentiation program: ((osteogenesis) OR (osteogenic)); ((adipogenesis) OR (adipogenic)); ((chondrogenesis) OR (chondrogenic)); ((odontogenesis) OR (odontogenic)); ((myogenesis) OR (myogenic)); ((cardiogenesis) OR (cardiogenic)); ((tenogenesis) OR (tenogenic)); ((neurogenesis) OR (neurogenic)).

**Figure 1:**
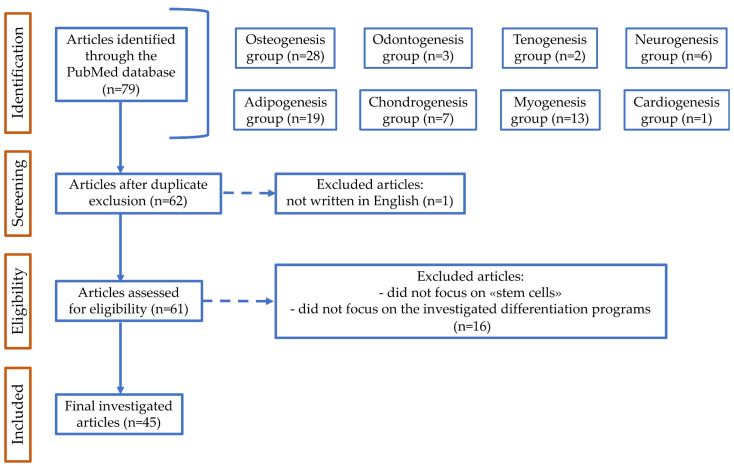
Flow diagram of PubMed data searching. n, number of articles identified, screened, excluded according to the declared criteria, and finally investigated.

To select more specific references, the following inclusion criteria were applied: (1) only original research articles; (2) written in English; and (3) no restrictions on the year of publication. The results obtained were then analyzed in order to exclude repeated references and to identify articles which fit the purpose of the research. The articles that did not directly focus on “stem cells” (or “mesenchymal stem cells” or “mesenchymal stromal cells”), cell differentiation, and cytochalasins were excluded. Finally, the selected articles were read and then, based on the topic, were assigned to the appropriate differentiation program/s. A comprehensive description of the search strategy and selection is available in Figure 1. 

## 3. Cytochalasins

Cytochalasins are a family of more than 60 different metabolites, produced by different species of fungi. Based on the size of the macrocyclic ring and the substitution of the perhydroisoindolyl-1-one residue located at the C-3 position, cytochalasins are classified into various subgroups [7,13]. The chemical diversity of each subgroup influences the biological properties of cytochalasins [13]. Cytochalasins were firstly isolated in the 1960s [7,14], and are named with the term “Phomin”, since they are isolated from the fungal species Phoma [14]; these molecules are also cytochalasins from the Greek words “cytos” (cells) and “chalasis” (relaxation), based on the observed effects of these compounds on mouse fibroblasts [15].

Cytochalasins inhibit actin polymerization and prevent microfilament assembly by binding both actin monomers and filaments [8,16]. Consequently, these mycotoxins, interfering with the cytoplasmatic actin organization, induce changes in cell morphology and affect cell division [13,17]. The chemical structure of each cytochalasin influences its specific functional properties and the possibility of having a reversible effect. For example, cytochalasin B (CB) or D (CD), widely used in research, are able to change cell morphology, and when they are removed from the culture medium, cells revert to their original shape [13,17].

CB is produced by the metabolic processes of Helminthosporium dematioideum and other fungi [18]. It inhibits actin polymerization by binding the fast-growing (barbed) end of F-actin, as well as by interacting with capping proteins (CAPZA1 and others in the F-actin capping protein α subunit family) [18,19]. CB is a well-known cytokinesis inhibitor which interferes with the contractile ring formation, as well as with the cleavage furrow development. Consequently, CB leads to the accumulation of multinucleated cells, as it blocks cell cytokinesis and does not affect the replication and division of the genetic material contained in the nucleus [13,15]. CB has other peculiar functions: (i) it can act as a glucose uptake inhibitor by competing with glucose itself in the binding of the plasma membrane transporters [20]; (ii) it can prevent the endocytosis process, leading to malabsorption of lipoproteins [21]; and (iii) it can alter cell migration by changing the cytoskeletal pattern as well as by interfering with the cytosolic Ca^2+^ storage [22].

CD is an isomeric metabolite of CB, derived from Metarrhizium anisopliae and Zygosporium mansonii [23]. Compared to CB, CD shows differences in the positioning of an ester group and the addition of a ketone group, as well as a contrast in the placement of double bonds. This different chemical structure makes CD much more potent than CB in preventing actin polymerization [24,25]. Thanks to its action on the cytoskeleton, CD can activate tumor suppressor protein 53 (p53)-dependent pathways, causing cell cycle arrest at the G1–S transition [26]. Furthermore, CD has been identified as a promising chemotherapeutic agent. Indeed, by blocking cytokinesis, CD prevents the division of neoplastic cells without interfering with DNA synthesis and karyokinesis [13]. 

Finally, several studies demonstrated that cytochalasins can influence differentiation processes. In the next sections, we will report the main findings related to the effects of CB and CD on the modulation of SC differentiation with particular attention to human MSCs (hMSCs), isolated from different sources, as well as to SCs recovered from other species.

## 4. Human Mesenchymal Stem Cells 

The term “MSCs” is an acronym used to describe a population of multipotent stem/progenitor cells located in the stromal component of several tissues and commonly referred to “mesenchymal stem cells”, “multipotent stromal cells”, “mesenchymal stromal cells”, and mesenchymal progenitor cells. MSCs represent an important tool in regenerative medicine due to their ability to promote tissue repair and homeostasis following injury [27,28]. 

hMSCs have specific characteristics defined by the International Society for Cellular Therapy (ISCT). According to the ISCT criteria, hMSCs show a fibroblast-like morphology and adhere to the plastic support when grown under standard conditions in vitro. In addition, they are positive for the cluster differentiation (CD) 105, CD73, and CD90 surface markers, while they express low levels of major histocompatibility complex (MHC) class I and are negative for MHC class II, CD11b, CD34, CD14, CD45, and CD31 markers. Although the majority of the MSC population exhibits this immune phenotype, recent studies have revealed that it can change based on tissue sources and localization [29]. Moreover, hMSCs differentiate in vitro in various cell types of mesodermal origin, such as osteocytes, adipocytes, and chondrocytes [30]. To date, it is known that hMSCs can differentiate even into non-mesenchymal cell types, like skin cells, nervous cells, hepatocytes, and cardiomyocytes [27,28].

Although bone marrow represents the main source of hMSCs, MSCs can be isolated from various tissues, including adipose tissue [31], dental pulp [32], yellow ligament [33], umbilical cord blood and Wharton’s jelly [34], and the placenta and fetal membranes [35]. Compared to the hMSCs isolated from adult sources, the ones isolated from fetal and perinatal tissues show an increased ability to proliferate and differentiate, as well as a prolonged in vitro lifespan before replicative senescence occurs [36]. Moreover, hMSCs isolated from different tissues show biological heterogeneity in their differentiation potential, being able to differentiate or not into a specific cell type [37]. Several investigations have also highlighted a reduction in stemness maintenance, proliferation rate, lifespan, and differentiation potential in hMSCs obtained from elderly donors, indicating that donor age as well as harvesting time may represent a critical factor which must be taken into consideration during MSC transplantation [38,39]. 

Finally, hMSCs exert their regenerative potential both directly and indirectly in organ and tissue repair [4,40] by restoring tissue homeostasis through paracrine effects that mediate cell-to-cell signaling, reducing local inflammation and increasing cell proliferation during tissue regeneration [41,42].

## 5. Cytochalasins and Osteogenesis

### 5.1. Effects of Cytochalasins on Cytoskeletal Organization and Cell Morphology during Osteogenesis

Osteogenic differentiation is a cellular process that involves specific cellular changes at both the structural and molecular level. During this process, SCs lose their typical fibroblast-like morphology and acquire an angular shape with a greater extension and size. These changes are due to a cytoskeletal structure modification, which involves the reorganization of actin microfilaments, leading to a switch from parallel-oriented distribution extending across the entire cytoplasm to a cortical location, as well as to an increase in actin polymerization and in the number of microfilaments [43,44].

Several research groups independently observed that CD inhibited hMSC osteogenic differentiation. In fact, CD-treated cells showed a reduction in calcium deposition and in the expression of alkaline phosphatase (ALP), RUNX family transcription factor 2 (RUNX2), and osteocalcin (OCN) [43,44,45,46]. Moreover, in a study by Sonowal and colleagues, it was reported that in human bone marrow MSCs (hBM-MSCs) a three-day CD treatment (100–1000 ng/mL) was sufficient to reduce osteogenic differentiation; indeed, CD affected the cytoskeletal actin polymerization, promoting a decrease in the phosphorylation levels of p38 mitogen-activated protein kinases (p38MAPKs) and in the expression of osteogenic-related genes, thus highlighting a fundamental role of actin organization in the induction of the osteogenic process [44].

Peng and colleagues used CD to study the relationship between actin filaments and MSC osteogenic differentiation induced by cyclic tensile stress. The authors demonstrated that cyclic tensile stress promoted the early stage of osteogenic differentiation in MSCs via integrin αVβ3 activation, leading to the rearrangement of the actin filaments, an increase in focal adhesion complex (FAC) formation, and Yes1-Associated Transcriptional Regulator (YAP) nuclear localization. CD treatment inhibited the effects elicited by the cyclic tensile stress, reducing the aggregation of actin filaments, nuclear YAP localization, and osteogenic marker expression [47].

To deepen the role of the cytoskeleton during the osteogenic process, Fan and colleagues demonstrated that in human adipose-derived stem cells (hASCs), CD (0.1 μg/mL) negatively affected osteogenesis by acting on the spatial organization and on the crosstalk between actin and vimentin, which are key players in the osteogenic process [48]. Actin and vimentin interact with the nucleus by binding to two nuclear proteins, Nesprin-3 and Nesprin-1/2, respectively. The expression of these proteins is modulated during osteogenic differentiation: vimentin and Nesprin-3 decrease during the osteogenic process, while the expression of actin and Nesprin-1/2 increase in the early stage of the differentiation. Consequently, the vimentin network becomes smaller and denser, localizing itself in a smaller area of the cytoplasm, while Nesprin-3, which is normally aggregated around the nucleus, spreads into the cytoplasm. On the other hand, actin filaments become thicker and multilayered on the ventral cell side, replacing the space left empty by vimentin, and as a result, cells increase their stiffness [48]. In addition, the authors demonstrated a vimentin-mediated control on actin expression, capable of modulating the osteogenic process. They showed that when vimentin expression was upregulated, there was a concomitant decrease in the expression of actin and Nesprin-1/2, and, therefore, in the cell stiffness, resulting in a reduction in *RUNX2* and *osteopontin* (*OPN*) gene levels [48]. In this context, the addition of CD to the osteogenic medium reduced the expression of actin filaments and of the associated Nesprin-1/2 while increasing the expression of vimentin and Nesprin-3, reversing their mutual cellular distribution. These changes were associated with a decrease in the expression of osteogenesis-related genes such as *RUNX2* and *OPN* and with an increase in the expression of phospho-Smad family member 2/3 (Smad2/3) and adiponectin, important markers of the adipogenic commitment, suggesting that CD can modulate SC fate by impairing the balance between actin and vimentin expression [48]. 

Pampanella and colleagues studied the effects of another cytochalasin, CB (0.01–5 μM), on osteogenic differentiation in human Wharton’s jelly MSCs (hWJ-MSCs), a perinatal MSC model [49]. The authors observed that CB-treated cells became more enlarged, with large focal adhesion regions connecting the stress fibers to the plastic support. Moreover, CB changed the mechanical properties of hWJ-MSCs by increasing actin bundles. In particular, at the highest CB concentration, hWJ-MSCs showed an increased stiffness in the peripheral area, probably due to the maintenance and strengthening of actin tension, which was localized at the cortical level. Moreover, vimentin supported both the cytoplasm and the nucleus and formed intense filament clusters, especially at the perinuclear level, which could maintain the structural integrity of the nucleus, counteracting cytoskeletal deformations and maintaining its biological functions. The overall reorganization of the cytoskeleton induced by CB favored osteogenic differentiation in a dose-dependent manner. In fact, CB treatment increased the expression of the osteogenic genes *RUNX2* and *OCN*, as well as the formation of calcium deposits [49]. Therefore, Pampanella and colleagues came to different conclusions than those previously discussed for CD, indicating that different cytochalasins may induce different effects on the same SC commitment, probably depending on the type of SCs treated [9,13].

CD can also modulate the cell spreading area, a parameter which influences the osteogenic process. Yao and colleagues, for example, demonstrated that in rat BM-MSCs (rBM-MSCs), osteogenesis was promoted by a high cell aspect ratio, which represents a measure of the stretching of a cell. CD treatment (0.25 mg/mL), by reducing the aspect ratio, induced alterations in cell morphology, leading to a reduction in the osteogenic potential of rBM-MSCs [50]. 

The ability of CD to change the cell spreading area and morphology was also investigated by Zhao’s group [51]. The authors cultured mouse BM-MSCs (mBM-MSCs) in substrates of different shapes (circular or star-like micropatterned substrates) with different spreading areas (1256 or 314 mm^2^) in the presence or absence of CD (1 μg/mL). The data evidenced that mBM-MSCs cultured in a 1256 mm^2^ star-like pattern showed a high density in actin filaments and stress fibers at the peripheral and central regions of the cells. On the contrary, when cells were cultured in the circular-pattern substrate of 314 mm^2^, the density of actin filaments decreased. The different cytoskeletal organization influenced the osteogenic differentiation, which was more pronounced in mBM-MSCs cultured in the star-like pattern of 1256 mm^2^ compared to those cultured in the other substrate. Such observations were confirmed by the increase in the expression of the *ALP*, *collagen type I* (*Col I*), and *OCN* genes. Moreover, mBM-MSCs cultured in a star-like pattern and in a higher spreading area expressed a higher level of β-catenin and showed an increase in their nuclear/cytoplasm ratio. It is known that, during osteogenic differentiation, β-catenin translocates from the cytoplasm to the nucleus, where it functions as a key player in the Wnt signaling pathway. The authors also demonstrated that the CD treatment of mBM-MSCs, by altering the organization and the assembly of actin, abrogated the difference in actin density and orientation between the two investigated pattern conditions, reducing the expression of osteogenic genes in both patterns, thus affecting the osteogenic process. In addition, CD reduced β-catenin levels and nuclear translocation, indicating that the mycotoxin may also impair the osteogenic process by modulating the activity of the Wnt pathway [51].

In another study, Xu and colleagues demonstrated that the manipulation of mASCs’ seeding densities resulted in large and small cells with distinguished microenvironments associated with actin cytoskeletal tension [52]. mASCs seeded at low and medium densities spread into large cells and showed robust osteogenesis, while mASCs at a high density were smaller and were differentiated into adipocytes. CD, by blocking the arrangement of actin cytoskeletal tension, induced the reorganization of cytoskeletal proteins influencing mASCs’ size and tension and, consequently, cell lineage differentiation. CD completely blocked adipogenesis in the presence of an adipogenic medium, yet inverted the osteogenic effects related to cell size in an osteogenic medium. CD reduced mineralization in low-density-seeded large cells, while it increased mineralization in high-density-seeded small cells [52].

### 5.2. Effects of Cytochalasins on Cell–Extra-Cellular Matrix Interactions during Osteogenesis

It is known that, during the early stages of cell commitment, the microenvironment may affect the differentiation potential of SCs. Extra-cellular matrix (ECM) properties such as matrix spatial organization, composition, and stiffness have been reported to be important regulators of osteogenic commitment [44]. 

The ECM and the intra-cellular actin filaments are closely connected to each other through a variety of cytoskeletal linker proteins; thus, the ability of cytochalasins to change the cytoskeleton architecture clearly influences the mechanical connection between intra-cellular and extra-cellular structures. 

Meka and colleagues observed that when hBM-MSCs were cultured on a 3D nanofibrous scaffold, which exhibits an architecture similar to the natural ECM, cells started to differentiate into osteocytes even in the absence of a specific osteogenic medium [53]. On this 3D substrate, hBM-MSCs showed distinct cell and nuclear morphologies, with lower areas and perimeters than cells grown on a flat two-dimensional (2D) substrate, but with higher aspect ratios. These changes were associated with a peculiar organization and localization of the actin microfilaments and microtubules that favored the osteogenic commitment. When the actin organization was impaired by using a CD treatment (1 μM), the osteogenic differentiation of the hBM-MSCs grown on the 3D scaffold decreased, as evidenced by a reduction in bone mineralization, a redistribution of heterochromatin, and a decrease in osteogenic gene expression. CD’s inhibition of osteogenesis was associated with a remodeling of cell morphology and nuclear shape that impaired the cell–ECM interactions [53]. 

Hu and colleagues also confirmed the ability of CD to interfere with cell–substrate interactions. Firstly, they studied the role of the surface topography in cell fate modulation using human periodontal ligament SCs (hPDLSCs) [54]. Cells cultured on a titanium surface expressed high levels of adhesion-related genes (*integrin β1* and *focal adhesion kinase*—*FAK*), improving their adhesion to the substrate, as well as increased stress fiber production. In this culture condition, hPDLSCs also showed an increase in the expression of osteogenic-related genes and proteins and a downregulation of adipogenic markers. Moreover, an increase in the nuclear translocation and transcriptional activity of the PDZ-binding motif (TAZ) transcription regulator was observed, revealing a contribution of the TAZ activity in the osteogenic process. In the same culture condition, CD treatment (0.2 μM), by interfering with the actin cytoskeleton and cell–substrate interactions, reduced TAZ expression and translocation into the nucleus, leading to a reduction in osteogenesis [54].

On the other hand, Keller and colleagues, studying the role of various ECM components on the modulation of the osteogenic process, showed that CD treatment (2 μM) modulated hASC osteogenic differentiation independently from the presence of specific ECM components [55]. In fact, CD treatment alone increased the expression of osteogenic markers and calcium deposition. On the contrary, ECM components alone seemed to be insufficient to guide a complete cell commitment, but they stimulated the osteogenic process by inducing changes in cell morphology. Interestingly, in the presence of both CD and fibronectin, hASCs still maintained an elongated shape, which favored the osteogenic process, despite the presence of CD, which, alone, stimulated the cells to assume a rounded shape; in these culture conditions, cells showed the highest increase in the expression of osteogenic transcription factors and markers, indicating that a strong relationship exists between the ECM, actin organization, and cellular morphology in the regulation of osteogenic differentiation [55].

Guo and colleagues tried to clarify whether cell morphology modulated cell differentiation by acting simultaneously or downstream of the matrix stiffness [56]. Firstly, the authors evaluated how cell morphology impacted cell commitment by culturing rBM-MSCs in the presence of fibronectin. They demonstrated that fibronectin (whose concentration correlates with matrix stiffness) enhanced F-actin assembly and modified cell morphology in a dose-dependent manner; such modifications enhanced the cell spreading area and promoted the osteogenic differentiation. The molecular mechanism behind the changes in F-actin organization and cell morphology involved the fibronectin-mediated translocation of YAP/TAZ proteins into the nucleus, which favored osteogenic gene expression. Later, to investigate the interplay between cell morphology and matrix stiffness, rBM-MSCs were cultivated in a soft or rigid matrix, fixing the cell spreading area to prevent changes in cell morphology. In these conditions, the authors observed that the matrix stiffness did not significantly affect YAP/TAZ activity and failed to modulate cell differentiation. All these findings indicated that cell morphology could be a downstream mediator of matrix stiffness-induced osteogenic differentiation [56].

Moreover, the authors demonstrated that CD (1 μM) alone reduced the osteogenic potential of rBM-MSCs. In fact, the mycotoxin significantly inhibited cell spreading and induced the cells’ round shape, promoting a reduction in *OCN* and *RUNX2* gene levels. On the contrary, a higher concentration of fibronectin in the presence of CD supplementation promoted an osteogenic program. In fact, fibronectin rescued cell spreading, counteracting changes in cell morphology induced by the mycotoxin, as also demonstrated by Keller and colleagues [55]. Therefore, in this study, fibronectin reversed CD’s osteogenic inhibition, mainly acting on cell morphology, a key player in osteogenic commitment [56].

### 5.3. Effects of Cytochalasins on the Cytoplasmic/Nuclear Actin Ratio in the Regulation of Osteogenesis

Different authors have speculated on the importance of actin nuclear trafficking in the modulation of osteogenic commitment. In fact, besides its mechanical functions in the cytoplasm, actin plays an important role in the nucleus, where it can exist in both monomeric and polymeric forms and can interact with multiple transcription factors, thus modulating the expression of several genes involved in osteogenic differentiation [57].

Sen and colleagues observed that, in hBM-MSCs and mBM-MSCs, CD supplementation (0.1 μg/mL) for 3 days induced a persistent actin fiber depolymerization and led to the translocation of actin monomers into the nucleus, thus increasing nuclear actin levels [57]. Such an increase promoted the cytoplasmic translocation of YAP, a transcription factor which acts in the nucleus inhibiting the expression of *RUNX2*, thus favoring an increase in the expression of osteogenesis-related genes. These molecular mechanisms were confirmed by blocking the translocation of actin towards the nucleus, which, in turn, prevents the translocation of YAP in the cytoplasm and affects CD-induced osteogenesis [57].

Wang and colleagues also observed a cytoplasmic translocation of YAP when they studied the effects of CD (0.2 μg/mL) in hPDLSCs. The translocation was associated with an increase in YAP’s phosphorylated form and a decrease in the expression of its target genes (*connective tissue growth factor* (*CTCG*) and *TEA domain* (*TEAD*) family members). However, unlike what is reported by Sen and colleagues, these authors described a CD downregulation of osteogenesis by reducing the expression of ALP, RUNX2, and OSX [58]. These contradictory results could be explained by the fact that cytochalasins can behave in a different way depending on the investigated SC type [49].

In another study, Sen and colleagues observed that, in mBM-MSCs, CD (0.1 mg/mL) not only destroyed F-actin filaments in single monomers and dimers, which translocated into the nucleus, but they also found that, inside the nucleus, monomeric actin started to assemble in filaments, which acted by modulating the chromatin architecture, thus influencing mBM-MSC fate. The formation of a primary actin filament was favored by CD through the transport of diaphanous-related formin 1 (mDia1) in the nucleus, where it co-worked with diaphanous-related formin 2 (mDia2). Then, both formins recruited the actin-related protein 2/3 (Arp2/3) complex, which initiated the second filament branching, a crucial step in starting osteogenic differentiation. In support of this, the inhibition of this complex formation abrogated the CD-induced osteogenesis and stimulated the cells to differentiate into adipocytes [59]. 

In line with these observations, Samsonraj’s group demonstrated in another cellular model, hASCs, that CD (0.1 μg/mL) stimulated osteogenesis by enhancing nuclear actin transport and the expression of genes coding for ECM proteins, cell surface receptors, cell adhesion molecules, and proteins involved in cell communication and signal transduction [60]. Some of these genes are involved in the osteogenic program, such as *HSD11B1*, which encodes the glucocorticoid-activating enzyme hydroxysteroid 11-beta dehydrogenase. A polymorphism in this gene has been associated with age-related osteoporosis and bone metabolism. Another gene upregulated by CD treatment was the *metastasis suppressor protein 1* (*MTSS1*), which is implicated in actin reorganization and in the regulation of cell motility by modulating different Arp2/3 activators. In parallel, CD decreased the expression of genes involved in nuclear processes and mitosis. One of these genes encodes an epigenetic regulator, the Polycomb Group 2 (PcG2) protein EZH2 (Enhancer of Zeste 2), a critical suppressor of osteoblastogenesis. An inverse correlation between EZH2 and RUNX2 was observed, suggesting that CD may regulate cell commitment by modulating the expression of key epigenetic regulators. Moreover, CD promoted the downregulation of TEAD4, a transcriptional factor able to interact with YAP and TAZ factors, controlling cell differentiation [60]. In another study [61], Samsonraj and colleagues compared the effect of CD (0.1 μg/mL) on gene expression in three MSC types, hASCs and h- and mBM-MSCs. Eight genes involved in cell adhesion, angiogenesis, and skeletal tissue development were commonly upregulated in all three types of MSCs. Among them, *Vestigial-like Family Member 4* (*VGLL4*), encoding for a co-regulator of the YAP/TAZ pathway, was upregulated when cells were cultured in the presence of CD. When *VGLL4* was silenced in hASCs, cells had a reduced ALP activity and mineralization process, indicating that CD enhanced the osteogenic differentiation of MSCs by modulating *VGLL4* expression [61].

**Figure 2:**
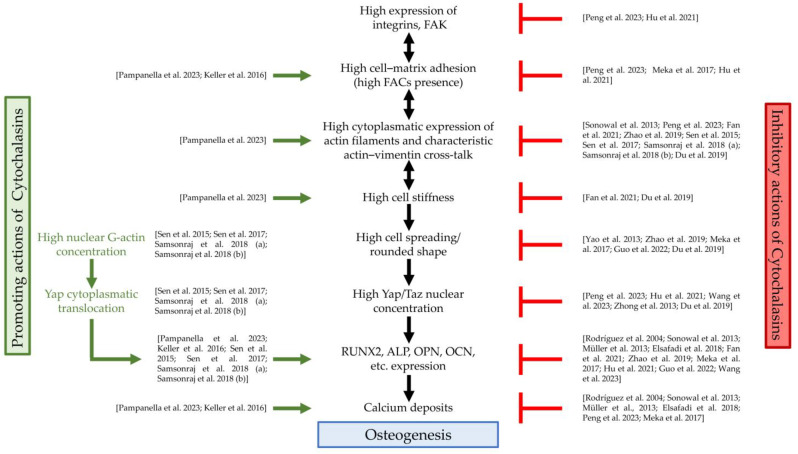
Cytochalasin actions and osteogenesis. FAK, focal adhesion kinase; FACs, focal adhesion complexes; Yap, Yes1-Associated Transcriptional Regulator; Taz, PDZ-binding motif (TAZ) transcription regulator; RUNX2, RUNX family transcription factor 2; ALP, alkaline phosphatase; OPN, osteopontin; OCN, osteocalcin; green arrows: actions of cytochalasins that promote osteogenesis; red symbols: actions of cytochalasins that counteract osteogenesis; references: Pampanella et al., 2023, [49]; Keller et al., 2016, [55]; Sen et al., 2015, [57]; Sen et al., 2017, [59]; Samsonraj et al., 2018 (a), [60]; Samsonraj et al., 2018 (b), [61]; Peng et al., 2023, [47]; Hu et al., 2021, [54]; Meka et al., 2017, [53]; Sonowal et al., 2013, [44]; Fan et al., 2021, [48]; Zhao et al., 2019, [51]; Du et al., 2019, [63]; Yao et al., 2013, [50]; Guo et al., 2022, [56]; Wang et al., 2023, [58]; Zhong et al., 2013, [62]; Rodríguez et al., 2004, [43]; Müller et al., 2013, [45]; Elsafadi et al., 2018, [46].

Table 1 summarizes the main studies and highlights the promoting or inhibiting effects of cytochalasins on SC osteogenesis, focusing on the treated cell type, as well as on the cytochalasin doses and type. Instead, Figure 2 shows, based on the literature evidence, at which level of the osteogenic pathway cytochalasins exert their functions.

**Table 1:** Cytochalasins and SC osteogenesis.

Reference Number	Title	Species and Cell Type	Molecular Treatment	Results *
[43]	Cytoskeletal Organization of Human Mesenchymal Stem Cells (MSC) Changes During Their Osteogenic Differentiation	hBM-MSCs	CD (0.1 μg/mL and 0.25 μg/mL)	↓
[44]	Inhibition of actin polymerization decreases osteogeneic differentiation of mesenchymal stem cells through p38 MAPK pathway	hBM-MSCs	CD (100–1000 ng/mL)	↓
[45]	Modulating the Actin Cytoskeleton Affects Mechanically Induced Signal Transduction and Differentiation in Mesenchymal Stem Cells	hBM-MSCs	CD (0.5 μM)	↓
[46]	TGF*β*1-Induced Differentiation of Human Bone Marrow-Derived MSCs Is Mediated by Changes to the Actin Cytoskeleton	hBM-MSCs	CD (not specified)	↓
[47]	Regulation of the integrin αVβ3-actin filaments axis in early osteogenic differentiation of human mesenchymal stem cells under cyclic tensile stress	hBM-MSCs hADSCs	CD (0.2 μg/mL)	↓
[48]	Spatial organization and crosstalk of vimentin and actin stress fibers regulate the osteogenic differentiation of human adipose-derived stem cells	hASCs	CD (0.1 μg/mL)	↓
[49]	Cytochalasin B Influences Cytoskeletal Organization and Osteogenic Potential of Human Wharton’s Jelly Mesenchymal Stem Cells	hWJ-MSCs	CB (0.01–5 μM)	↑
[50]	Effects of aspect ratios of stem cells on lineage commitments with and without induction media	rBM-MSCs	CD (0.25 mg/mL)	↓
[51]	Spreading Shape and Area Regulate the Osteogenesis of Mesenchymal Stem Cells	mBM-MSCs	CD (1 μg/mL)	↓
[52]	Connective Tissue Growth Factor in Regulation of RhoA Mediated Cytoskeletal Tension Associated Osteogenesis of Mouse Adipose-Derived Stromal Cells	mASCs	CD (0.5 μg/mL)	(related to seeding density)
[53]	Role of Microtubules in Osteogenic Differentiation of Mesenchymal Stem Cells on 3D Nanofibrous Scaffolds	hBM-MSCs	CD (1 μM)	↓
[54]	The Role and Activation Mechanism of TAZ in Hierarchical Microgroove/Nanopore Topography-Mediated Regulation of Stem Cell Differentiation	hPDLSCs	CD (0.2 μM)	↓
[55]	Correlation between ECM guidance and actin polymerization on osteogenic differentiation of human adipose-derived stem cells	hASCs	CD (2 μM)	↑
[56]	Relationship of matrix stiffness and cell morphology in regulation of osteogenesis and adipogenesis of BMSCs	rBM-MSCs	CD (1 μM)	↓
[57]	Intranuclear Actin Regulates Osteogenesis	hBM-MSCs mBM-MSCs	CD (0.1 μg/mL)	↑
[58]	LRP6/filamentous-actin signaling facilitates osteogenic commitment in mechanically induced periodontal ligament stem cells	hPDLSCs	CD (0.2 μg/mL)	↓
[59]	Intranuclear Actin Structure Modulates Mesenchymal Stem Cell Differentiation	mBM-MSCs	CD (0.1 mg/mL)	↑
[60]	Osteogenic Stimulation of Human Adipose-Derived Mesenchymal Stem Cells Using a Fungal Metabolite That Suppresses the Polycomb Group Protein EZH2	hASCs	CD (0.1 μg/mL)	↑
[61]	Validation of Osteogenic Properties of Cytochalasin D by High-Resolution RNA-Sequencing in Mesenchymal Stem Cells Derived from Bone Marrow and Adipose Tissues	hASCs hBM-MSCs mBM-MSCs	CD (0.1 μg/mL)	↑
[62] ^#1^	Mesenchymal stem cell and chondrocyte fates in a multishear microdevice are regulated by Yes-associated protein	MSCs	CD (not specified)	↓
[63] ^#2^	Topographic cues of a novel bilayered scaffold modulate dental pulp stem cells differentiation by regulating YAP signalling through cytoskeleton adjustments	hDPSCs	CD (1 μg/mL)	↓

h, human; r, rat; m, mouse; BM-MSCs, bone marrow mesenchymal stem cells; DPSCs, dental pulp-derived stem cells; ASCs or ADSCs, adipose-derived stem cells; PDLSCs, periodontal ligament-derived mesenchymal stem cells; WJ-MSCs, Wharton’s jelly mesenchymal stem cells; CD, cytochalasin D; CB, cytochalasin B; * arrows in the Results column indicate the effect of cytochalasins on SC osteogenesis: ↓ (reduce), ↑ (promote). ^#1^ Manuscript results are described in “Section 7”. ^#2^ Manuscript results are described in “Section 8.1”.

## 6. Cytochalasins and Adipogenesis

The adipogenic commitment in MSCs requires the upregulation of both early and late adipogenic gene markers, including *peroxisome proliferator-activated receptor gamma* (*PPARγ*), *adiponectin* (*ADIPOQ*), *lipoprotein lipase* (*LPL*), and *fatty acid-binding protein 4* (*FABP4*). Moreover, during the in vitro differentiation, cells are positive in Oil Red O solution (which stains the lipid vesicles red) [64] and change their morphology, becoming more rounded and less stiff with an increase in cytoplasmic monomeric G-actin and a decrease in assembled F-actin. In fact, adipogenesis is associated with a reduction in the organization and tension of the actin cytoskeleton [64]. In addition, this cellular commitment is promoted by a reduction in the substrate stiffness and in the interaction between the cell membrane and the ECM [56,65], correlated with a lower expression of RhoA/Rho-associated protein kinase (ROCK) and FAK [54,64,66]. FAK and RhoA/ROCK stabilize the cytoskeleton, while their inhibition makes cells softer [66].

Several studies on SCs treated with CD or CB have demonstrated that these mycotoxins, by affecting the actin cytoskeleton, modify cell morphology, thus modulating the adipogenic program [67]. In particular, mycotoxin-treated hMSCs showed reduced cell spreading and the loss of their fibroblast-like shape, cell area, and aspect ratio [44,45,50,56,68]. Changes in cell morphology were linked to a dose-dependent destruction of actin fiber organization [54,68] due to the breakage and depolymerization of actin filaments [69], followed by a translocation of G-actin in the nucleus, where this monomer is able to interfere with gene expression [59]. Moreover, in response to higher concentrations of toxins, actin filaments lost their colocalization with vinculin [45], abolishing its canonic distribution pattern, thus promoting the disappearance of FACs [68]. The overall changes in the cytoskeleton led to a reduction in cell stiffness, as demonstrated by analyzing two nanomechanical properties, the elasticity and viscosity, with atomic force microscopy (AFM) in CB-treated hASCs. In these cells, CB reduced the elasticity and increased the viscosity, shifting their behavior towards that of a pure liquid material [68]. Moreover, when CB was removed, the cells rescued their cytoskeletal organization and cell morphology, as well as cell stiffness, highlighting the reversible action of this mycotoxin [17,68].

All of the effects of cytochalasins on MSCs described above favor adipogenic commitment. In fact, cells treated with CD or CB enhanced their expression of adipogenic markers such as PPARγ [46,68,69], CCAAT Enhancer Binding Protein Alpha (C/EBPα) [67,68], LPL [46,69], ADIPOQ [57], and FABP4 [69], as well as increasing the number of lipid vesicles [45,46,64,66,67,68]. Furthermore, cytochalasins promoted the rotation of the nucleus and a change in the polarization of actin filaments, two typical cellular events that occur in early adipogenic commitment and guide the early pattern of tissue morphogenesis [70]. In fact, while hMSCs cultured in a standard medium exhibited a positive cell orientation and anticlockwise (ACW)-biased nucleus rotation associated with the swirling of actin filaments, cytochalasin treatment reinforced the effects of adipogenic induction: the cell chiral orientation became negative, and the nucleus rotation, as well as actin swirling, became CW-biased [70].

Moreover, the CD effect on actin depolymerization modified several molecular pathways involved in the adipogenic program. For example, CD treatment promoted a decreased expression of FAK, RhoA/ROCK, and Smad2. The latter is a transcriptional modulator which, when active, represses C/EBPα activation; thus, its reduced expression favors the adipogenic ability of MSCs [64,66]. In addition, CD, by inhibiting RhoA/ROCK and FAK activities, promoted the phosphorylation of p38 and extra-cellular signal-regulated kinase ½ (ERK1/2), which play a crucial positive role in MSC adipogenesis [66]. Finally, CD enhanced adipogenesis and inhibited osteogenesis by regulating the expression of key candidate genes, including *Fibroblast growth factor 2* (*FGF2*), *Transforming growth factor-beta 2* (*TGFβ2*), *Plasminogen Activator*, *Tissue Type* (*Plat*), *Early Growth Response 2* (*EGR2*), *Myocyte enhancer factor 2D* (*MEF2D)*, and *Insulin receptor substrate 1* (*IRS1*) [46].

These data indicate that both CD and CB enhance adipogenesis commitment regardless of cell types and toxin doses.

**Figure 3:**
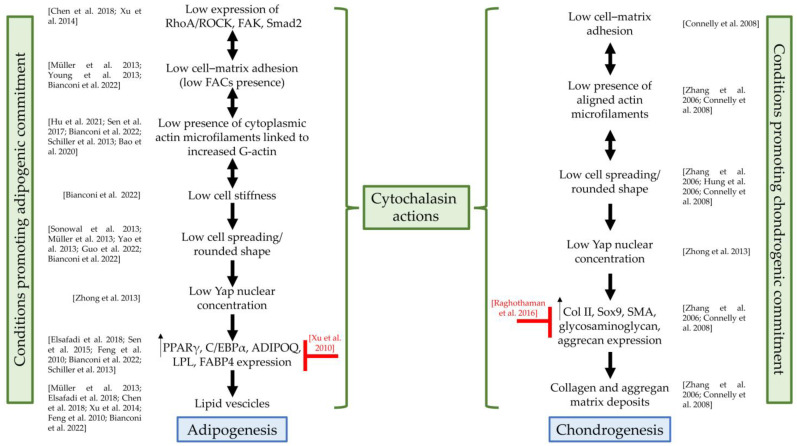
Cytochalasin actions on adipogenesis and chondrogenesis. RhoA/ROCK, RhoA/Rho-associated protein kinase; FAK, focal adhesion kinase; Smad2, phospho-Smad family member 2; FACs, focal adhesion complexes; Yap, Yes1-Associated Transcriptional Regulator; PPARγ, peroxisome proliferator-activated receptor gamma; C/EBPα, CCAAT Enhancer Binding Protein Alpha; ADIPOQ, adiponectin; LPL, lipoprotein lipase; FABP4, fatty acid-binding protein 4; Col II, collagen II; Sox9, SRY-Box Transcription Factor 9; SMA, alpha-smooth muscle actin; green curly brackets: actions of cytochalasins that promote adipogenesis or chondrogenesis; red symbols: actions of cytochalasins that counteract differentiation programs; references: Chen et al., 2018, [64]; Xu et al., 2014, [66]; Müller et al., 2013, [45]; Young et al., 2013, [65]; Bianconi et al., 2022, [68]; Hu et al., 2021, [54]; Sen et al., 2017, [59]; Schiller et al., 2013, [69]; Bao et al., 2020, [70]; Sonowal et al., 2013, [44]; Yao et al., 2013, [50]; Guo et al., 2022, [56]; Zhong et al., 2013, [62]; Elsafadi et al., 2018, [46]; Sen et al., 2015, [57]; Feng et al., 2010, [67]; Xu et al., 2010, [52]; Connelly et al., 2008, [71]; Zhang et al., 2006, [72]; Hung et al., 2006, [73]; Raghothaman et al., 2016, [74].

Table 2 summarizes the main studies and highlights the promoting or inhibiting effects of cytochalasins on SC adipogenesis, focusing on the treated cell type, as well as on the cytochalasin doses and type. Furthermore, Figure 3 shows, based on the literature evidence, at which level of the adipogenic and chondrogenic pathways cytochalasins exert their functions.

## 7. Cytochalasins and Chondrogenesis

The chondrogenic commitment in MSCs requires the production of collagen II (Col II), SRY-Box Transcription Factor 9 (Sox9, a transcription factor for Col II), glycosaminoglycan, and aggrecan. Once synthesized, these products are poured into the ECM, being detected in vitro with Alcian blue staining. Moreover, chondrogenesis is associated with morphological changes in SCs, becoming more rounded, and with a reduction in cell–matrix interactions. These changes reflect the dynamic remodeling of actin filaments [71,72,73]. Finally, the chondrogenic differentiation of MSCs was favored in vitro by the presence of specific molecules in the culture medium. TGFβ1, for example, induces a dose-dependent increase in glycosaminoglycan and Col II synthesis, as well as in the expression of alpha-smooth muscle actin (SMA). In vitro, SMA-positive cells mimic those located within the surface layer of articular cartilage, which could play a significant role in cartilage development and maintenance [73].

Cytochalasins, altering the F-actin cytoskeleton and modifying cell–matrix or cell–cell interactions, influence chondrogenic commitment [71,72,73,74]. Published data revealed conflicting results about the role of these toxins in chondrogenic differentiation.

For example, Zhang and colleagues demonstrated that CD supplementation favored a chondrogenesis program in SCs derived from a murine embryoid body (EB) [72]. They treated SCs with CD (0.5–10 μg/mL) which was added to both a basal culture medium and a chondrogenic medium. In both conditions, the mycotoxin led cells to acquire a round shape and the actin filaments appeared to be distributed in the peripheral region of the cytoplasm. Moreover, when CD was added to the chondrogenic medium, the SCs showed an upregulation of the expression of chondrogenic gene markers within 5 days of treatment compared to cells cultured only in the chondrogenic medium. CD promoted a complete chondrogenic process by inducing a significant downregulation in the expression of undifferentiation/stemness markers (*octamer-binding transcription factor 4* (*Oct4*) and *Sox2*), as well as of ectodermal, endodermal, and mesodermal differentiation/cardiogenesis representative markers, such as *Sox1*, *alpha fetoprotein* (*AFP*), and *cardiac troponin-1* (*CTN-1*), respectively [72].

Hung and colleagues demonstrated that a higher CD concentration had a cytotoxic effect on SCs. In fact, by adding CD at the concentration of 10 μM in the TGFβ1 chondrogenic medium, the mycotoxin led to an increase in the number of apoptotic hBM-MSCs. Moreover, in this condition, CD did not affect SMA expression and chondrogenic protein synthesis, but only altered the cell morphology and their capacity to adhere to the plastic culture support [73].

The differentiation of MSCs into chondrocytes is also influenced by different microenvironmental niches. By investigating the role of the cell–substrate interactions in chondrogenesis, Connelly and colleagues demonstrated that the supplementation of the chondrogenic medium with CD (0.3 μM) in a calf BM-MSC (cBM-MSC) culture reduced the cell adhesion in a three-dimensional agarose hydrogel (modified with synthetic peptides containing arginine–glycine–aspartic acid, RGD), thus inducing a decrease in cell spreading and an increase in the production of sulfated glycosaminoglycan (sGAG), a marker of chondrogenesis [71].

An opposite role of CD in chondrogenesis emerged in a recent study by Raghothaman and colleagues [74]. The authors demonstrated that cell–cell interactions are required to achieve a robust MSC chondrogenesis. To this end, hMSCs encapsulated in an interfacial polyelectrolyte complexation (IPC)-based hydrogel containing Col I differentiated toward chondrogenesis, resulting in mature hyaline neocartilage tissue characterized by the expression of aggrecan, Col II, cartilage oligomeric matrix protein (COMP), and Col IX. On the contrary, the supplementation of CD (0.5 μM) promoted a reduction in cell–cell interactions, abrogating cell elongation and promoting a single-cell-like state. Consequently, Col II expression was downregulated and the chondrogenic differentiation of hMSCs was reduced, indicating a negative role of CD in this condition [74].

Finally, Zhong and colleagues demonstrated that CD influenced the expression and localization of YAP protein that, as well as for other SC commitments, was important in the chondrogenic differentiation. The authors pointed out that an increase in YAP expression promoted chondrocyte dedifferentiation, while a CD-dependent reduction in YAP, nuclear accumulation favored the maintenance of the chondrocyte phenotype, as well as MSC adipogenesis, negatively affecting the osteogenic commitment [62].

Table 3 summarizes the main studies and highlights the promoting or inhibiting effects of cytochalasins on SC chondrogenesis, focusing on the treated cell type, as well as on the cytochalasin doses and type.

## 8. Cytochalasins and Other Mesodermal Differentiation Commitments

### 8.1. Odontogenesis

Human dental pulp-derived stem cells (hDPSCs) are a population of MSCs isolated from adult dental pulp tissue. They express a pattern of surface markers similar to the other hMSCs, but compared to these cells, they exhibit higher clonogenic abilities and growth rate. Due to their easy surgical access, high proliferation, and multilineage potential, hDPSCs are considered a promising candidate for regenerative medicine approaches. They are especially transplanted to enhance the regeneration of the dentin–pulp complex in damaged tissues through the odontogenic differentiation program [75]. Odontogenesis is associated with a reorganization of the cytoskeleton characterized by a high presence of tick actin stress fiber bundles and focal adhesions, able to confer cellular stiffness. Moreover, in this differentiation, an increase in mineralized deposits and a predominantly nuclear localization of YAP is evident [75,76,77].

Several factors have been shown to promote the differentiation ability of hDPSCs toward odontogenic commitment, but CD has been shown to counteract their effects. For example, Zheng and colleagues demonstrated that the application of a one-millitesla (mT) static magnetic field for 24 h increased the density of the actin cytoskeleton, the hDPSC osteo/odontogenic capacity (as demonstrated by the increased expression of both ALP and dentin sialophosphoprotein (DSPP), osteogenic and odontogenic markers, respectively), and mineralization [77]. Moreover, the magnetic field recruited YAP and TAZ to the nucleus, inhibited their phosphorylation, and upregulated the expression of the *connective tissue growth factor* (*CTGF*) and *ankyrin repeat domain 1* (*ANKRD1*) genes, known regulators of YAP/TAZ proteins [77].

Moreover, Du and colleagues reported that hDPSC odontogenic differentiation was also enhanced by specific culture supports, such as the bilayered poly lactic-co-glycolic acid (PLGA) scaffold used. In this condition, hDPSCs improved F-actin stress fiber alignment, and the spreading cell area showing an elongated cell appearance and a nuclear localization of YAP [63].

CD counteracted the effects exerted by both the static magnetic field and the PLGA scaffold in hDPSCs. This mycotoxin reduced the presence and the alignment of actin stress fibers, cell stiffness, and YAP/TAZ nuclear localization [63,77] with a change in morphology towards a more rounded shape [76]. Moreover, cytoplasm-restricted YAP localization reduced the expression of Collagen type I alpha 1 chain (COLIA1), an early odontogenic marker, and inhibited the mineralization of DPSC cultures [77]. All together, these results demonstrate the inhibitory role of CD in odontogenesis.

### 8.2. Myogenesis

Myogenic differentiation requires myoblast migration, high cell density, cell–cell interactions, and syncytium formation by myoblasts [78,79,80,81,82]. In vitro, C2 mouse myoblasts, undifferentiated cells capable of differentiating into myocytes, and their subclone C2C12 are often used as a model to study this process [78].

During the migration of C2C12 myoblasts into a wounded monolayer, centrosomes are oriented in the direction of migration and the nucleus has a rearward movement correlated with that of actin retrograde flow. Chang and colleagues demonstrated that treatment with CD (0.5 µM) of C2C12 myoblasts prevented centrosome orientation and the movement of the nucleus, thus affecting myoblast migration, which was found to be an actin-dependent process [78].

In a study by Balogh and colleagues, it was demonstrated that treatment with CD (1 µg/mL for 30 h) influenced myogenesis in vitro, also by perturbing cell–cell contacts and, consequently, the cell density. A high culture density of C2 myoblasts is an important condition for promoting the stable expression of stathmin, a cytoplasmic phosphoprotein, whose expression is strongly regulated during tissue development as it controls the proliferation of cells and their entry into the myogenic process. Treatment with CD, preventing high cell density through its action on the actin cytoskeleton, induced a reduction in stathmin expression, thus affecting the differentiation of C2C12 myoblasts [79].

Formigli and colleagues demonstrated that treatment with dihydrocytochalasin B (DHCB, 1 µg/mL), a cytochalasin sharing with the other mycotoxins its actin-disrupting action, induced the alteration of the functional interaction between actin stress fibers and stretch-activated channels (SACs) [80]. In mouse C2C12 cell myogenic differentiation, the formation of stress fibers and their contraction generate a mechanical tension at the plasma membrane which, by activating SACs, influences the maturation of myoblasts into myotubes. Formigli’s group demonstrated that DHCB, acting on the cytoskeletal/SAC interaction, inhibited the expression of myogenic markers (myogenin and sarcomeric proteins) and the myoblast–myotube transition, thus affecting mouse C2C12 cell myogenic differentiation [80].

In vitro myogenesis also involves the formation of syncytia between myoblasts. To better mimic postnatal myogenesis, Knepper and colleagues co-cultured myotubes with undifferentiated myoblasts, observing 48 h after the start of co-culture the occurrence of postnatal myonuclear accretion. The presence of CD (0.3 µM) in the co-culture resulted in a decreased postnatal myonuclear growth, also highlighting the importance of the actin cytoskeleton in syncytia formation [81].

Finally, it is known that myoblast differentiation is influenced by the expression of multiple specific factors, such as heterochromatin protein 1 gamma (HP1γ). Charò and colleagues demonstrated that HP1γ allowed myoblasts to differentiate into thin myotubes, colocalizing with actin in both the cytoplasm and nucleus. In the cytoplasm, HP1γ was associated with the perinuclear actin cap, which controls the shape and position of the nucleus. In the nucleus, HP1γ-actin was associated with the promoter and the transcribed regions of the housekeeping gene *GAPDH*, suggesting that HP1γ may function as a scaffold protein for the recruitment of actin to control gene expression. Furthermore, the authors demonstrated that HP1γ was able to co-immunoprecipitate with actin in myoblasts incubated with or without CD (1 µg/mL), which, by depolymerizing the actin, reduced the presence of F-actin, indicating that HP1γ plays its role being able to interact with both F- and G-actin. Therefore, since CD does not interfere with the promoting action of HP1γ in myogenesis, this means that this mycotoxin uses other molecular pathways when it negatively controls the myogenic process [82].

In conclusion, the overall results highlight a prevalent inhibitory role of cytochalasins on myoblast differentiation.

### 8.3. Tenogenesis

It is known that hBM-MSCs have a multilineage differentiation potential. Among the different cell types, they can differentiate into tenocytes and can be used for tendon/ligament repair [83] The differentiation of these cells into tendon/ligament-like lineages requires an elongated cell shape, a high density of actin fibers with a perpendicular alignment, as well as FAK phosphorylation, and a high expression of genes and proteins encoding for Col I, Col III, tenascin C (TNC), scleraxis BHLH transcription factor (SCX), ephrin type-A receptor 4 (epha4), EYA transcriptional coactivator and phosphatase 2 (eya2), and SIX homeobox 1 (six1) [84].

In a study by Xu and colleagues, it was demonstrated that treatment of BM-MSCs with CD (1 μg/mL) for 1 h before mechanical stretching counteracted its tenogenic effects by acting at both the cytoskeletal and molecular level. In fact, the cells became rounded, and the organization of actin fibers was disrupted, affecting the alignment of the cells and actin fibers with respect to the direction of stretch. Moreover, a decrease in FAK phosphorylation and a marked reduction in the expression levels of the most relevant mechanical stretch-induced genes and proteins were observed, suggesting an inhibitory role of CD on hBM-MSC tenogenesis, a differentiation process that requires the integrity of cytoskeletal organization [84].

Table S1 summarizes the main studies and the effects of cytochalasins on other mesodermal differentiations, focusing on the treated cell type, as well as on the cytochalasin doses and type.

## 9. Cytochalasins and Neurogenesis

Neurogenesis, like other differentiation processes, is favored by both cell–cell and cell–substrate interactions and is influenced by cytoskeletal organization and cell morphology [85,86,87,88].

Laplante and colleagues demonstrated that a treatment of P19 mouse embryonic stem cells (mESCs) with CB (5 μM) had no direct effect on the aggregation and expression of cadherin, a protein involved in cell–cell interactions, and, consequently, on neurogenesis [85]. On the contrary, the differentiation of mESCs into neurons was significantly decreased when the cells were treated with colchicine, an agent capable of destroying microtubules and reducing the expression of cadherin, a positive and key player of neurogenesis [85].

Czeiler and colleagues instead provided evidence about the relevance of cell–substrate interactions in neurogenesis, showing that CD-induced migratory reactions and morphological changes in embryonic neural stem cells (NSCs) were influenced by different in vitro topographical scaffolds that mimicked the in vivo niches [86]. In detail, large-fiber topography without laminin, which mimics the topography of blood vessels, prevented NSC migration and reduced the morphological changes induced by a treatment with 0.1 μM of CD. On the other hand, CD did not influence cell morphology when cells were cultured in the presence of small-fiber topography (made to mimic radial glial process topography). These data indicate that cell morphology depends on different cytoskeletal proteins (not only actin on which CD acts) and on the topographical environment in which SCs grow. In fact, the physical structure of distinct scaffolds induces unique signaling cascades that regulate migration and morphology during neurogenesis. Therefore, CD does not act alone on neurogenesis, but by integrating its action with that of other factors [86].

To deepen the role of actin cytoskeleton on neurogenesis, Kronenberg and colleagues treated gelsolin-deficient (Gsn^−/−^) synaptosomes with CD (1 μM) which, like gelsolin, produces actin disassembly [87]. Gelsolin is widely expressed into the brain of adult mammalians and its silencing reduces actin turnover, eliciting a strong increase in the hippocampal neurogenesis of (Gsn^−/−^) mouse, as demonstrated by an increase in depolarization-induced calcium levels, which promoted a greater exocytotic release of norepinephrine in presynaptic terminals. A treatment with CD confirmed that the positive effects of gelsolin deficiency on neurogenesis were directly attributable to impaired actin filament turnover. In fact, CD induced in (Gsn^−/−^) synaptosomes a decrease in Ca^2+^ influx and subsequent exocytotic norepinephrine release, suggesting that this mycotoxin represents a negative modulator of hippocampal neurogenesis in vivo [87].

CD (1 μM) was also used by Ohara and colleagues to study the mechanism of axon formation. They demonstrated that the organization of the neurite growth cones was comparable between rat hippocampal neurons and human induced pluripotent stem cell (hiPSC)-derived neurons during the very early developmental stage. CD induced the translocation of drebrin and F-actin from the transitional zone to the distal edge of the growth cones in both neurons, as well as promoting the same distribution of microtubules that extended into the edge of the growth cone, although the axon growth was slower in hiPSC-derived neurons [88].

Neurogenesis was also influenced by the concentration of glucose in the culture medium and its uptake in the cell. Sawangmake and colleagues demonstrated the deleterious effects of high extra-cellular glucose concentrations during neurosphere formation. These effects were confirmed by culturing hPDLSCs in a neurogenic culture medium supplemented with CB (10 μM), also known to be an inhibitor of glucose uptake [20,89]. In fact, CB induced in the treated cells an accumulation of extra-cellular glucose, which negatively affected the neurosphere size and the expression of neurogenic markers, confirming the role of CB in preventing glucose uptake and highlighting the negative effect of high extra-cellular glucose concentrations in neurosphere formation [89].

In conclusion, cytochalasins modulate the neurogenic program by acting directly on actin organization and cell morphology, as well as on cell–substrate interactions, being involved in several molecular pathways and often showing inhibitory effects on neurogenesis.

Table S2 summarizes the main studies and the effects of cytochalasins on neurogenesis, focusing on the treated cell type, as well as on the cytochalasin doses and type.

## 10. Conclusions

In conclusion, in this review, we have shown that cytochalasins can modulate the differentiation potential of SCs by reorganizing the actin cytoskeleton, by inducing changes in cell morphology and stiffness, and by modulating the cell–cell and cell–substrate interactions. The use of cytochalasins displays some advantages. These compounds have a sustainable cost and are easy to handle for in vitro treatments. On the other hand, it is evident that the effects of cytochalasins, at both the structural and molecular levels, depend on the toxin dose and type, as well as on the cellular model used. In detail, low concentrations of cytochalasins facilitate the study of the mechanisms underlying stem cell commitment or could be used in vitro to commit stem cells before in vivo regenerative approaches. On the contrary, high concentrations of these mycotoxins (>10 μM) could exert cytotoxic effects on specific stem cell types, inducing apoptosis [73,90] or the cell proliferation block [49,68], thus affecting the possibility of cell expansion in vitro. Failure to identify an adequate dose of cytochalasin in relation to the cellular model investigated could be a limit in the use of these compounds both in vitro and in vivo. Moreover, the discordant results obtained by different authors make difficult to define the effects of different cytochalasins on a specific differentiation program. Nevertheless, the observed discrepancies may reflect the complexity of a pleiotropic action of cytochalasins (such as, for example, on the actin cytoskeleton or cellular glucose uptake), unfolding into specific differentiating outcomes on the basis of multifaceted context/microenvironmental cues that could hardly be standardized from one laboratory to another worldwide. Probably, the attainment of more homogenous, consensus-based strategies of cell culture and scaffolding conditions, as well as the systematic analysis of the effects of specific cytochalasins as a function of the tissue source of hMSCs, may provide additional, less contradictory insights into the use of these molecules for steering the directionality of their multilineage rescuing potential.

## Figures and Tables

**Table 2:** Cytochalasins and SC adipogenesis.

Reference Number	Title	Species and Cell Type	Molecular Treatment	Results *
[64]	Actin depolymerization enhances adipogenic differentiation in human stromal stem cells	hBM-MSCs	CD (1–20 μM)	↑
[56]	Relationship of matrix stiffness and cell morphology in regulation of osteogenesis and adipogenesis of BMSCs	rBM-MSCs	CD (1 μM)	↑
[65]	Stimulation of adipogenesis of adult adipose-derived stem cells using substrates that mimic the stiffness of adipose tissue	hASCs	CD (0.25 μg/mL)	↑
[54]	The Role and Activation Mechanism of TAZ in Hierarchical Microgroove/Nanopore Topography-Mediated Regulation of Stem Cell Differentiation	hPDLSCs	CD (0.2 μM)	↑
[66]	Role of p38, ERK1/2, focal adhesion kinase, RhoA/ROCK and cytoskeleton in the adipogenesis of human mesenchymal stem cells	hMSCs	CD (0.02 μg/mL)	↑
[67]	Cytoskeletal Disassembly and cell rounding promotes adipogenesis from ES cells	mESCs	CD (0.2–20 μM)	↑
[44]	Inhibition of actin polymerization decreases osteogeneic differentiation of mesenchymal stem cells through p38 MAPK pathway	hBM-MSCs	CD (100–1000 ng/mL)	↑
[45]	Modulating the Actin Cytoskeleton Affects Mechanically Induced Signal Transduction and Differentiation in Mesenchymal Stem Cells	hBM-MSCs	CD (0.5 μM)	↑
[50]	Effects of aspect ratios of stem cells on lineage commitments with and without induction media	rBM-MSCs	CD (0.25 mg/mL)	↑
[68]	Cytochalasin B Modulates Nanomechanical Patterning and Fate in Human Adipose-Derived Stem Cells	hASCs	CB (1–10 μM)	↑
[69]	Adipogenesis of adipose-derived stem cells may be regulated via the cytoskeleton at physiological oxygen levels in vitro	hASCs	CD (2 μM)	↑
[59]	Intranuclear Actin Structure Modulates Mesenchymal Stem Cell Differentiation	mBM-MSCs	CD (0.1 mg/mL)	↑
[46]	TGF*β*1-Induced Differentiation of Human Bone Marrow-Derived MSCs Is Mediated by Changes to the Actin Cytoskeleton	hBM-MSCs	CD (not specified)	↑
[57]	Intranuclear Actin Regulates Osteogenesis	hBM-MSCs mBM-MSCs	CD (0.1 μg/mL)	↑
[70]	Early Committed Clockwise Cell Chirality Upregulates Adipogenic Differentiation of Mesenchymal Stem Cells	hMSCs	CD (0.02–0.2 μM)	↑
[52] ^#1^	Connective Tissue Growth Factor in Regulation of RhoA Mediated Cytoskeletal Tension Associated Osteogenesis of Mouse Adipose-Derived Stromal Cells	mASCs	CD (0.5 μg/mL)	↓
[62] ^#2^	Mesenchymal stem cell and chondrocyte fates in a multishear microdevice are regulated by Yes-associated protein	MSCs	CD (not specified)	↑

m, mouse; h, human; r, rat; ESCs, embryonal stem cells; BM-MSCs, bone marrow mesenchymal stem cells; ASCs, adipose-derived stem cells; PDLSCs, periodontal ligament-derived mesenchymal stem cells; CD, cytochalasin D; CB, cytochalasin B; * arrows in the Results column indicate the effect of cytochalasins on SC adipogenesis: ↓ (reduce), ↑ (promote). ^#1^ Manuscript results are described in “Section 5.1.”. ^#2^ Manuscript results are described in “Section 7”.

**Table 3:** Cytochalasins and SC chondrogenesis.

Reference Number	Title	Species and Cell Type	Molecular Treatment	Results *
[72]	Reorganization of actin filaments enhances chondrogenic differentiation of cells derived from murine embryonic stem cells	mEBCs	CD (0.5–10 μg/mL)	↑
[73]	Alpha-smooth muscle actin expression and structure integrity in chondrogenesis of human mesenchymal stem cells	hBM-MSCs	CD (10 μM)	cytotoxic dose
[71]	Interactions Between Integrin Ligand Density and Cytoskeletal Integrity Regulate BMSC Chondrogenesis	cBM-MSCs	CD (0.3 μM)	↑
[74]	Cell type dependent morphological adaptation in polyelectrolyte hydrogels governs chondrogenic fate	hBM-MSCs	CD (0.5 μM)	↓
[62] ^$^	Mesenchymal stem cell and chondrocyte fates in a multishear microdevice are regulated by Yes-associated protein	MSCs	CD (not specified)	↑

m, mouse; h, human; c, calf; EBCs, embryoid body-derived cells; BM-MSCs, bone marrow mesenchymal stem cells; CD, cytochalasin D; * arrows in the Results column indicate the effect of cytochalasins on SC chondrogenesis: ↓ (reduce), ↑ (promote); ^$^ article retrieved in adipogenic and osteogenic searches but also related to chondrogenesis.

## Supplementary Materials

The following supporting information can be downloaded at https://www.mdpi.com/article/10.3390/cells13050400/s1: Table S1. Cytochalasins and mesodermal differentiation; Table S2. Cytochalasins and neurogenesis.
