# Supplementary material for: Cytochalasins as Modulators of Stem Cell Differentiation
Source: Cells. 2024 Feb 25;13(5):400. doi: 10.3390/cells13050400 (PMC10931372; doi:10.3390/cells13050400)
Supplement: Supplementary file 1 [file cells-13-00400-s001.zip › cells-2860637-supplementary.pdf]

Table S1. Cytochalasins and mesodermal differentiation.

	Reference Number	Title	Species and Cell Type	Molecular Treatment	Results*
**Odontogenesis**	[76]	Mechanical Changes in Human Dental Pulp Stem Cells during Early Odontogenic Differentiation	hDPSCs	CD (5 μM)	↓
	[77]	Static magnetic field regulates proliferation, migration, differentiation, and YAP/TAZ activation of human dental pulp stem cells	hDPSCs	CD (10 μM)	↓
	[63]	Topographic cues of a novel bilayered scaffold modulate dental pulp stem cells differentiation by regulating YAP signalling through cytoskeleton adjustments	hDPSCs	CD (1 μg/mL)	↓
**Myogenesis**	[78]	Linker of nucleoskeleton and cytoskeleton (LINC) complex-mediated actin-dependent nuclear positioning orients centrosomes in migrating myoblasts	mC2C12 myoblasts	CD (0.5 μM)	↓
	[79]	Growth and cell density-dependent expression of stathmin in C2 myoblasts in culture	mC2 myoblasts	CD (1 μg/mL)	↓
	[80]	Cytoskeleton/stretch-activated ion channel interaction regulates myogenic differentiation of skeletal myoblasts	mC2C12 myoblasts	DHCB (1 μg/mL)	↓
	[81]	A novel in vitro model for the assessment of postnatal myonuclear accretion	mC2C12 myoblasts	CD (0.3 μM)	↓
	[82]	Heterochromatin protein (HP)1γ is not only in the nucleus but also in the cytoplasm interacting with actin in both cell compartments	mC2C12 myoblasts	CD (1 μg/mL)	↓
**Tenogenesis**	[84]	RhoA/ROCK, cytoskeletal dynamics, and focal adhesion kinase are required for mechanical stretch-induced tenogenic differentiation of human mesenchymal stem cells	hBM-MSCs	CD (1 μg/mL)	↓

h, human; m, mouse; DPSCs, dental pulp-derived stem cells; C2, C2 mouse myoblasts; C2C12, subclone of C2 mouse myoblasts; bone marrow mesenchymal stem cells, BM-MSCs; RhoA/Rho-associated protein kinase, RhoA/ROCK; cytochalasin D, CD; dihydrocytochalasin B, DHCB; *arrows in the results column indicate the effect of cytochalasins on mesodermic differentiation: ↓ (reduce).

Table S2. Cytochalasins and neurogenesis.

Reference Number	Title	Species and Cell Type	Molecular Treatment	Results*
[85]	RhoA/ROCK and Cdc42 regulate cell-cell contact and N-cadherin protein level during neurodetermination of P19 embryonal stem cells	P19 mESCs	CB (5 μM)	-
[86]	Surface topography during neural stem cell differentiation regulates cell migration and cell morphology	h/mNSCs	CD (0.1 μM)	(related to the surface topography)
[87]	Impact of actin filament stabilization on adult hippocampal and olfactory bulb neurogenesis	mouse synaptosomes	CD (1 μM)	↓
[88]	Early-stage development of human induced pluripotent stem cell-derived neurons	rat neurons hiPSC-derived neurons	CD (1 μM)	-
[89]	High glucose condition suppresses neurosphere formation by human periodontal ligament-derived mesenchymal stem cells	hPDLSCs	CB (10 μM)	↓

h, human; m, mouse; ESCs, embryonic stem cells; PDLSCs, periodontal ligament-derived mesenchymal stem cells; iPSC, induced pluripotent stem cell; CB, NSCs, neural stem cells; cytochalasin B; CD, cytochalasin D; *arrows in the results column indicate the effect of cytochalasins on neurogenesis: - (no effect) ↓ (reduce).
